# Estimating species pools for a single ecological assemblage

**DOI:** 10.1186/s12898-017-0155-7

**Published:** 2017-12-22

**Authors:** Tsung-Jen Shen, Youhua Chen, You-Fang Chen

**Affiliations:** 1 Institute of Statistics & Department of Applied Mathematics, National Chung Hsing University, 250 Kuo Kuang Road, Taichung, 40227 Taiwan; 2 Chengdu Institute of Biology, Chinese Academy of Sciences, Chengdu, 610041 China; 3 Department of Renewable Resources, University of Alberta, Edmonton, AB T6G 2H1 Canada; 4 School of Software, Harbin Normal University, Harbin, China

**Keywords:** Regional processes, Distributional aggregation, Sampling theory, Jackknife estimator, Unseen species, Asymptotic variance

## Abstract

**Background:**

The species pool concept was formulated over the past several decades and has since played an important role in explaining multi-scale ecological patterns. Previous statistical methods were developed to identify species pools based on broad-scale species range maps or community similarity computed from data collected from many areas. No statistical method is available for estimating species pools for a single local community (sampling area size may be very small as ≤ 1 km^2^). In this study, based on limited local abundance information, we developed a simple method to estimate the area size and richness of a species pool for a local ecological community. The method involves two steps. In the first step, parameters from a truncated negative trinomial model characterizing the distributional aggregation of all species (i.e., non-random species distribution) in the local community were estimated. In the second step, we assume that the unseen species in the local community are most likely the rare species, only found in the remaining part of the species pool, and vice versa, if the remaining portion of the pool was surveyed and was contrasted with the sampled area. Therefore, we can estimate the area size of the pool, as long as an abundance threshold for defining rare species is given. Since the size of the pool is dependent on the rarity threshold, to unanimously determine the pool size, we developed an optimal method to delineate the rarity threshold based on the balance of the changing rates of species absence probabilities in the sampled and unsampled areas of the pool.

**Results:**

For a 50 ha (0.5 km^2^) forest plot in the Barro Colorado Island of central Panama, our model predicted that the local, if not regional, species pool for the 0.5 km^2^ forest plot was nearly the entire island. Accordingly, tree species richness in this pool was estimated as around 360. When the sampling size was smaller, the upper bound of the 95% confidence interval could reach 418, which was very close to the flora record of tree richness for the island. A numerical test further demonstrated the power and reliability of the proposed method, as the true values of area size and species richness for the hypothetical species pool have been well covered by the 95% confidence intervals of the true values.

**Conclusions:**

Our method fills the knowledge gap on estimating species pools for a single local ecological assemblage with little information. The method is statistically robust and independent of sampling size, as proved by both empirical and numerical tests.

**Electronic supplementary material:**

The online version of this article (10.1186/s12898-017-0155-7) contains supplementary material, which is available to authorized users.

## Background

Ecological communities are assembled from a variety of regional and local processes [1]. As a regional process, the species pool hypothesis has gained much attention in contemporary ecology over the past decades [2, 3]. The species pool hypothesis posits a group of species present in a larger area that is ready to colonize a local community [4–7]. Whereas the concept of species pool has been applied in empirical studies, it is still challenging to accurately determine the area size and contained species number of the species pool.

Species pool size is important for determining the space–time community structure of local samples, in both neutral and niche theories [6, 8–14]. To evaluate the statistical significance of regional process roles in species diversity patterns, some null models have been employed [15, 16]. However, the central problem accompanying these null models is adequate delineation of the species pool, which could strongly influence the interpretation of relevant mechanisms underpinning local species diversity. Previous delineation of the species pool was usually carried out in relatively arbitrary or empirical ways. For example, some studies define the size of species pool based on ecologically pertinent areas, such as biogeographic regions or terrestrial continents [2, 17].

Two types of spatial data have been widely employed in ecological studies: large-scale distribution of species and local-scale distribution of species. Some recent studies [2, 18–21] developed statistical methods to delineate the species pool, which typically require computing pairwise distance or species turnover [22]; or performing regression. Apparently, the performance of these methods is limited as they require many data gathered from many large-scale or mesoscale areas as inputs (e.g., range maps of species or species richness collected from a large number of sampling units). When only limited data (i.e., species abundance data) surveyed from a very local community are in hand, all of these methods would be unsuitable and inapplicable. Here a local community is defined to have a sampling area size no more than 1 km^2^ (e.g., permanent forest plots). Until now, there has been no statistical method available for delineating local or regional species pools [6, 12, 23] based on species distribution or abundance data from a single ecological community.

One difficulty in defining an adequate species pool is the compounding effect of species that are absent from the local community, or very rare and not accounted for during sampling despite being present [24, 25]. According to the definition of species pool, these unseen species are typically undocumented for the local community but certainly will be present in a larger community [26, 27]. These species may be detected by expanding the sampling domain to neighboring areas of the local plot. To this end, predicting the number of unseen species based on limited abundance information of observed species in the local community is a key to identify proper local or regional species pools for a single local community.

Defining the species pool should reflect the species spatial distribution. Species distribution is not random in space, usually presenting an aggregation pattern [28]. A regular pattern is also possible. Therefore, a statistical method for delineating a species pool should be able to describe these general species distributional patterns in both the local community and its pool. For achieving such a goal, a parametric probabilistic model accounting for distributional aggregation might be used. The negative binomial model (NBD) has been used extensively for modeling species distributional aggregation [29, 30], but it is not directly related to the areal size of the species pool. It should be modified when applied to model a species pool.

Two quantities need to be addressed when relating the concept of species pool to species diversity patterns in a local community: the areal size of the species pool and the number of species in the pool. For the available data provided from a specific local community, how can we estimate these two quantities? To achieve this goal, by (1) using some equivalence assumptions between unseen and rare species and (2) modeling distribution aggregation of species in the local community, we develop a simple probabilistic method to infer area size and species diversity of the local, if not regional, species pool for the local community.

Please note that species pool can be either local or regional in the early development of the concept [6, 23]. For a regional species pool, it is defined at a broad scale. However, for a local species pool, its spatial extent can be very small and local. Species in the local species pool can migrate into the targeted community in very short time and distance [6, 23]. In the later development of the concept, species pool is quantified in a more probabilistic and numerical way [18, 19], in which the delineation of species pool is a function of the migration ability of species in the targeted ecological community (this could also be applied to the statistical model in our paper). In this case, local and regional species pools represent a continuum of the overall dispersal ability of species across different spatial scales [5]. To this end, if the general dispersal ability of species in the targeted ecological community is low, the corresponding species pool for the targeted community is expected to be small, being a local species pool.

## Methods

### A truncated negative trinomial model

**Fig. 1:**
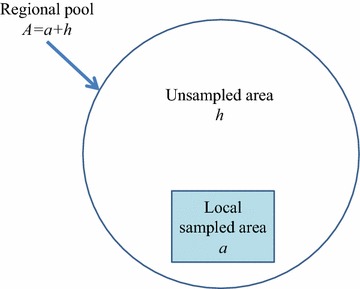
Spatial relationships between species pool *A*, local sampled (or censused) area *a*, and remaining unsampled area *h*. Note that *A* = *a* + *h*

Assume there are *S*_*A*_ species present over a large biogeographic region with area *A*. The region can be decomposed into two disjoint parts with respective areas *a* and *h* as in Fig. 1, where *a* is the sampled area and the whole region *A* represents its species pool.

Note that *A* = *a* + *h*. Let *X*_*a*_ and *X*_*h*_ denote the numbers of organisms of a species respectively scattered over the two parts. To account for the dependency of the two abundances in the areas *a* and *h* for the same species, we extend the NBD model to a truncated negative trinomial distribution (NTD) with the probability function as

1 $$\begin{aligned} P(X_{a} = x, \, X_{h} = y) & = \frac{\varGamma (\alpha + x + y)}{\varGamma (x + 1)\varGamma (y + 1)\varGamma (\alpha )} \\ & \quad \times \,\frac{{\left( {\frac{\beta }{\beta + A}} \right)^{\alpha } \left( {\left( {\frac{a}{\beta + A}} \right)^{x} \left( {\frac{h}{\beta + A}} \right)^{y} - I(x + y = 0)} \right)}}{{1 - \left( {1 + {A \mathord{\left/ {\vphantom {A \beta }} \right. \kern-0pt} \beta }} \right)^{ - \alpha } }} , { } \\ \end{aligned}$$

where *x* and *y* are nonnegative integers; *I*(·) is an indicator function and defined as *I*(*E*) = 1 if the statement *E* is true; otherwise *I*(*E*) = 0. The truncation of the model at zero is necessary to ensure that all considered species belonging to the species pool are present in the pool, otherwise *S*_*A*_ is undefined. All species are assumed to share the same parameters *α* and *β* because they inhabit the same region with similar environmental factors. The model parameter *α* is used to measure spatial distribution aggregation, while *β* is a rate parameter having a reciprocal relationship to the mean abundance. A further interpretation of Eq. (1) is provided in the Additional Methods of Additional file 1.

Note that, from the model in (**1**), the species abundance in the sampled area *X*_*a*_ can be proven to have a marginal probability function

2 $$P(X_{a} = x) = \frac{{\frac{\varGamma (\alpha + x)}{\varGamma (x + 1)\varGamma (\alpha )}\,\left( {\frac{\beta }{\beta + a}} \right)^{\alpha } \left( {\left( {\frac{a}{\beta + a}} \right)^{x} - \left( {\frac{\beta + a}{\beta + A}} \right)^{\alpha } I(x = 0)} \right)}}{{1 - \left( {1 + {A \mathord{\left/ {\vphantom {A \beta }} \right. \kern-0pt} \beta }} \right)^{ - \alpha } }}, \, x \ge 0,$$

### Estimation of parameters *α* and *β*

Let the sampled data be $\left( {Q_{ 1} ,Q_{ 2} , \ldots ,Q_{M} } \right)$ from the local area *a*, where *Q*_*n*_ represents the number of species with *n* individuals in the data. When the observed number of species in the sampled area *a* is given, $\left( {Q_{ 1} ,Q_{ 2} , \ldots ,Q_{M} } \right)$ follows a multinomial distribution with total $\sum\nolimits_{n = 1}^{M} {Q_{n} }$ and cell probabilities $\left( {\phi_{ 1} , \phi_{ 2} , \ldots , \phi_{M} } \right),$ where

3a $$\phi_{n} = \frac{{P(X_{a} = n)}}{{1 - P(X_{a} = 0)}}$$

and *M* is the maximum abundance observed in the local area *a*. Theorem 1 in Additional file 1 provides a proof for explaining why $\left( {Q_{ 1} ,Q_{ 2} , \ldots ,Q_{M} } \right)$ follows the multinomial distribution. As a result, the log likelihood function is expressed as follows:

3b $$L\left( {\alpha ,\beta \left| {Q_{1} , \ldots ,Q_{M} } \right.} \right) = C + \sum\limits_{n = 1}^{M} {Q_{n} \log \left( {\phi_{n} } \right),}$$

where *C* is a constant, which is unrelated to parameters *α* and *β*. Note that analogous applications can be found in previous studies [31, 32]. The maximum likelihood estimators (MLE) $\hat{\alpha }$ and $\hat{\beta }$ of *α* and *β* can be found by maximizing the log likelihood function, independent of the species pool area *A*.

### Estimation of the area size for the species pool

For a large species pool the unsampled area *h* in Fig. 1 would have many species that could not be observed in the sampled area *a*, and vice versa. These species may have species abundances 1, 2, *…*. in the unsampled area *h*. However, if a species is very common in *h*, it is very likely to be seen in *a* as well; the reverse is also true. Therefore, only those rare species in the unsampled region *h* (or sampled area *a*) with numbers of individuals less than a rarity threshold (e.g., 10) would be likely unseen in the sampled area *a* (or unsampled area *h*). These species thus constitute the candidate species unseen in the sampled area *a* (or unsampled area *h*). For computation feasibility, and since the size of *h* is unknown, the same threshold *t* is used for both the sampled and unsampled regions. Accordingly, the expected number of unseen species in the area *a* (or *h*) should have the form:

4 $$\left\{ \begin{aligned} E_{a} (Q_{0} ) = \sum\limits_{i = 1}^{t} {E_{h} (Q_{i} )} \hfill \\ E_{h} (Q_{0} ) = \sum\limits_{i = 1}^{t} {E_{a} (Q_{i} )} \hfill \\ \end{aligned} \right.,$$

where *t* is the population threshold for defining the rare species. Additionally, *E*_*a*_(*Q*_0_) and *E*_*h*_(*Q*_0_) denote the expected numbers of unseen species in the sampled area *a* and unsampled area *h*, respectively. *E*_*a*_(*Q*_*i*_) and *E*_*h*_(*Q*_*i*_) denote the expected numbers of species with abundance *i* in the sampled area *a* and unsampled area *h*, respectively, i.e., *E*_*a*_(*Q*_*i*_) = *S*_*A*_ *P*(*X*_*a*_ = *i*) and *E*_*h*_(*Q*_*i*_) = *S*_*A*_ *P*(*X*_*h*_ = *i*). Here *S*_*A*_ is thus far unknown, but is cancelled out when substituted into both sides of Eq. (4). Thus, the resultant equations are as follows:

5 $$\left\{ \begin{aligned} P(X_{a} = 0) = \sum\limits_{i = 1}^{t} {P(X_{h} = i)} \hfill \\ P(X_{h} = 0) = \sum\limits_{i = 1}^{t} {P(X_{a} = i)} \hfill \\ \end{aligned} \right..$$

For example, in a 50 ha (0.5 km^2^) forest plot from the Barro Colorado Island of central Panama (BCI) [33–36], a tree species has 696 living individuals on average based on 2005 census data. It is reasonable that species with abundances less than 10 or a larger value in unsampled habitat *h* would be unseen in the BCI plot. In this case, the boundary of *h* is unknown, and may include the remaining part of BCI island or neighboring mainland territory. Thus, *t* would be pre-defined as 10, then by inserting Eq. (2) into Eq. (3) or Eq. (4) when *α* and *β* have been estimated or given, we can estimate the size of unsampled area *h* or the size of species pool *A,* using *A* = *a* + *h* (Fig. 1).

When *t* = 1, we have *E*_*a*_(*Q*_0_) = *E*_*h*_(*Q*_1_) and *E*_*h*_(*Q*_0_) = *E*_*a*_(*Q*_1_), equivalently, *E*_*a*_(*Q*_0_)/*E*_*h*_(*Q*_1_) = 1 and *E*_*h*_(*Q*_0_)/*E*_*a*_(*Q*_1_) = 1. Thus, the number of unseen species in sampled area *a* can be estimated as the number of singletons in the remaining area *h*, or vice versa. This is similar to the first-order Jackknife estimator of species richness [37, 38].

To numerically solve *h* for a given population threshold *t*, following Eq. (5), we minimize the following quantity as

6 $$\left| {P(X_{a} = 0) - \sum\limits_{i = 1}^{t} {P(X_{h} = i)} } \right|^{2} + \;\left| {P(X_{h} = 0) - \sum\limits_{i = 1}^{t} {P(X_{a} = i)} } \right|^{2} .$$

In practice, when the population rarity threshold *t* is unknown and no empirical values can be referred to, it is necessary to define an optimal threshold *t* based on the limited species information from sampled area *a*. It is possible to establish another formula using the probability of a species unseen in the unsampled area, *P*(*X*_*h*_ = 0), decreasing in *h* while conversely, *P*(*X*_*a*_ = 0) is increasing. As a result, we consider an equilibrium status of unseen species in the species pool for which the increasing *P*(*X*_*h*_ = 0) rate is approximately equal to the decreasing *P*(*X*_*a*_ = 0) rate. Therefore, the optimal *t* can be numerically found from minimizing

7 $$\left| {\frac{\partial }{\partial A}P(X_{h} = 0) + \frac{\partial }{\partial A}P(X_{a} = 0)} \right|^{2} .$$

Numerically, for many given *t* values [and correspondingly many candidate *A* values solved from Eq. (6)], the optimal value should be the smallest, after which the square of the difference in Eq. (7) would change slowly (which can be clearly shown in the results). Here we set the optimal threshold to the largest *t* to make the square of the difference larger than 10^−10^.

### Estimation of species number for the species pool

If the optimal $\hat{t}$ and area size of pool $\hat{A}$ have been determined using Eqs. (6) and (7), we could estimate the species number $S_{{\hat{A}}}$ in the pool by solving the following equality as,

8 $$S_{{\hat{A}}} P(X_{a} \ge 1) = S_{{\hat{A}}} \left( {1 - P(X_{a} = 0)} \right) \approx S_{a} ,$$

where *S*_*a*_ is the number of observed species found in sampled area *a*. For the BCI forest plot, the 2005 census data have *S*_*a*_ = 298. Finally, *P*(*X*_*a*_) is related to the MLEs $\hat{\alpha }$ and $\hat{\beta }$. Solving Eq. (8), we obtain an explicit formula for estimating species number in the estimated pool $\hat{A}$ as,

9 $$S_{{\hat{A}}} = S_{a} \frac{{\left( {1 + \frac{{\hat{A}}}{{\hat{\beta }}}} \right)^{{\hat{\alpha }}} - 1}}{{\left( {1 + \frac{{\hat{A}}}{{\hat{\beta }}}} \right)^{{\hat{\alpha }}} - \;\left( {\frac{{\beta + \hat{A}}}{{\hat{\beta } + a}}} \right)^{{\hat{\alpha }}} }}.$$

### Asymptotic variances and 95% confidence interval for the area size and species number of the species pool

Because *A* = *a* + *h* and *a* are fixed, the variance of the estimated area size *A*, found from Eq. (6), is equal to the variance of estimated *h* (i.e., $Var(\hat{A}) = Var(\hat{h})$), which is computed by defining

$$G(h,\hat{\alpha },\hat{\beta }) = \left. {\left\{ {\left( {\left| {P(X_{a} = 0) - \sum\limits_{i = 1}^{t} {P(X_{h} = i)} } \right|^{2} } \right)^{{\frac{1}{2}}} + \left( {\left| {P(X_{h} = 0) - \sum\limits_{i = 1}^{t} {P(X_{a} = i)} } \right|^{2} } \right)^{{\frac{1}{2}}} } \right\}} \right|_{{(\alpha ,\beta ) = (\hat{\alpha },\hat{\beta })}} .$$

Use the Taylor expansion of $G(\hat{h},\hat{\alpha },\hat{\beta })$ at $\hat{h} = h$ (see Additional file 1 for details), we then approximate the variance of $\hat{h}$ using

10 $$Var(\hat{h}) \approx \left[ {\left. {\frac{\partial }{{\partial \hat{h}}}G(\hat{h},\hat{\alpha },\hat{\beta })} \right|_{{\hat{h} = h}} } \right]^{ - 2} Var\left( {G(h,\hat{\alpha },\hat{\beta })} \right).$$

For the variance of $S_{{\hat{A}}}$, we define $H(\hat{h},\hat{\alpha },\hat{\beta }) = {{S_{{\hat{A}}} } \mathord{\left/ {\vphantom {{S_{{\hat{A}}} } {S_{a} }}} \right. \kern-0pt} {S_{a} }}$, which only involves $\hat{h} = \hat{A} - a$, $\hat{\alpha }$, and $\hat{\beta }$ while it is unrelated to observed species richness *S*_*a*_ in the sampled area. Using the variance decomposition formula repeatedly, the variance of $S_{{\hat{A}}}$ can be estimated:

11 $$\begin{aligned} V\hat{a}r(S_{{\hat{A}}} ) & \approx S^{2}_{a} \left( {{\mathbf{v^{\prime}}}\varSigma^{ - 1} {\mathbf{v}} + \left. {\left( {\frac{\partial }{\partial h}H(h,\alpha ,\beta )} \right)^{2} } \right|_{{(h,\alpha ,\beta ) = (\hat{h},\hat{\alpha },\hat{\beta })}} V\hat{a}r\left( {\hat{h}} \right)} \right) \\ & \quad + \;H^{2} (\hat{h},\hat{\alpha },\hat{\beta })S_{a} \left( {1 - \frac{{S_{a} }}{{S_{{\hat{A}}} }}} \right). \\ \end{aligned}$$

The technical derivation of the above formulas (Eqs. 10 and 11) and definition of each symbol on the right side of the formulas for both $Var(\hat{A})$ and $V\hat{a}r(S_{{\hat{A}}} )$ have been presented in detail in Additional file 1.

A 95% confidence interval (CI) of the species pool *A* can be conventionally derived from a normality assumption. However, the resultant lower bound of the 95% CI of *A* could be smaller than the local sample area when *A* is considerably larger than *a*. To avoid this situation, we applied a log-transformation to the 95% CI of *A*. This technique has been applied to species richness estimation [39], and the details are provided as follows.

Assume that $\hat{h} = \hat{A} - a$ follows a log normal distribution, i.e., $\text{log}(\hat{h})$ is distributed normally, then the 95% CI of *A* is expressed by $\left[ {a + {{\hat{h}} \mathord{\left/ {\vphantom {{\hat{h}} {R_{A} }}} \right. \kern-0pt} {R_{A} }}, \, a + \hat{h} \times R_{A} } \right]$, where

12 $$R_{A} = \exp\left\{ {1.96\left[ {\log\left( {1 + {{Var(\hat{A})} \mathord{\left/ {\vphantom {{Var(\hat{A})} {\hat{h}^{2} }}} \right. \kern-0pt} {\hat{h}^{2} }}} \right)} \right]^{{{1 \mathord{\left/ {\vphantom {1 2}} \right. \kern-0pt} 2}}} } \right\}.$$

Note that the merit of the resultant 95% confidence interval is that the lower bound is always larger the sampled area *a*.

Similar to the derivation of a 95% CI of *A*, we assume that $S_{{\hat{A}}} - S_{a}$ follows a log normal distribution, thus the 95% CI of *S*_*A*_ is $\left[ {S_{a} + {{\left( {S_{{\hat{A}}} - S_{a} } \right)} \mathord{\left/ {\vphantom {{\left( {S_{{\hat{A}}} - S_{a} } \right)} R}} \right. \kern-0pt} R}_{S} , \, S_{a} + \left( {S_{{\hat{A}}} - S_{a} } \right) \times R_{S} } \right]$, where

13 $$R_{A} = \exp\left\{ {1.96\left[ {\log\left( {1 + {{Var\left( {S_{{\hat{A}}} } \right)} \mathord{\left/ {\vphantom {{Var\left( {S_{{\hat{A}}} } \right)} {\left( {S_{{\hat{A}}} - S_{a} } \right)^{2} }}} \right. \kern-0pt} {\left( {S_{{\hat{A}}} - S_{a} } \right)^{2} }}} \right)} \right]^{{{1 \mathord{\left/ {\vphantom {1 2}} \right. \kern-0pt} 2}}} } \right\}.$$

### An empirical test

In our study, the entire 50-ha BCI plot was investigated (sampling fraction = 1). In addition, tree communities from three smaller sampling areas were also studied separately for comparison, with sample fractions set at 0.25 (12.5 ha), 0.5 (25 ha) and 0.75 (37.5 ha), respectively. For each sampling size, we applied the truncated NTD model described above and its marginal distribution to determine the distributional aggregation status of all species in the local community. Then Eqs. (6, 7, and 9) were used to determine the optimal threshold of rarity, area size and species richness of the species pool. The 95% confidence intervals of the area and species richness of the pool were estimated using Eqs. (12, 13). All computations were conducted using R software [40] and the computational R code for implementing the proposed method for estimating species pools is available in Additional file 2.

### A numerical test

We also conducted a numerical test by setting a hypothetical species pool with area size *A* = 1500 ha and species number *S*_*A*_ = 2000. Given a local sample with size *a* = 60 and species frequency counts $\left( {Q_{ 1} ,Q_{ 2} , \ldots ,Q_{M} } \right)$ generated from the truncated NTD model with *α* = 0.1 and *β* = 1 using Eq. (2), we tested the performance of our proposed method on estimating the hypothetical species pool regarding its area size and the corresponding species richness (i.e., *A* = 1500 and *S*_*A*_ = 2000).

Being similar to the empirical test above, the above hypothetical local area *a* with size 60 was further divided into four different sampling scales (thus representing different sample sizes) for testing the robustness and scale insensitivity of the proposed method separately as *a* = 60, 45, 30 and 15. We then used Eq. (7) to determine the optimal threshold *t* value for each sample size; and the optimal result was displayed in Additional file 1: Figure S1. Given the optimal threshold *t* value identified for each sampling scale, 95% confidence intervals of *A* and *S*_*A*_ can be constructed again using Eqs. (12, 13) accordingly. Figs. S1–S3 of this numerical example can be reproduced step-by-step using the computational R code provided in Additional file 2.

## Results

**Fig. 2:**
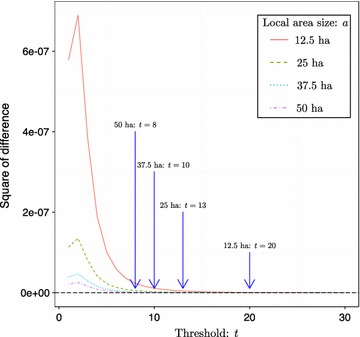
Square of the difference between the changing rates of unseen probabilities in the sampled area *a* and unsampled habitat *h*, for different threshold *t* values. We choose a cutoff point here as 10^−10^ (shown as the black horizontal dashed line), after which the square of the difference would approach zero and thus decrease very slowly. Different curves represent different sampling fractions (or local area size) of the entire BCI forest plot used to infer the area size of the species pool. The optimal threshold positions for different sampling sizes are highlighted with vertical arrows

By applying the optimal criterion using Eq. (7), we set the optimal threshold of *t* = 20, 13, 10 and 8 for the cases when sampling fractions were 0.25, 0.5, 0.75 and 1, respectively, in the BCI plot (Table 1; Fig. 2). By setting the above optimal threshold for each sampling size, the square of the difference in Eq. (7) would reduce slowly and reach a stable value closed to zero (Fig. 2).

**Table 1:** Estimated area size, species richness, and 95% confidence intervals of the species pool for the BCI forest plot

Sampling fraction	$\hat{\alpha }$	Area size (ha)	Optimal threshold *t*	Area size of pool *A*		Species richness of pool *S*_*A*_	
				Estimate	95% CI	Estimate	95% CI
0.25	0.075	12.5	20	1169.2	(843.0, 1623.5)	357.2	(319.0, 417.7)
0.50	0.088	25	13	1082.6	(736.2, 1597.7)	362.3	(335.8, 401.4)
0.75	0.105	37.5	10	1015.1	(604.8, 1722.3)	349.8	(325.8, 388.3)
1.0	0.100	50	8	973.5	(636.2, 1504.9)	359.8	(339.0, 391.7)

Results from the four different sampling fractions of the plot are presented and compared to show the estimated robustness of our model with respect to varying sampling sizes. Optimal rarity threshold *t* and clumping parameter $\hat{\alpha }$ for each sampling scale are also provided for reference

**Fig. 3:**
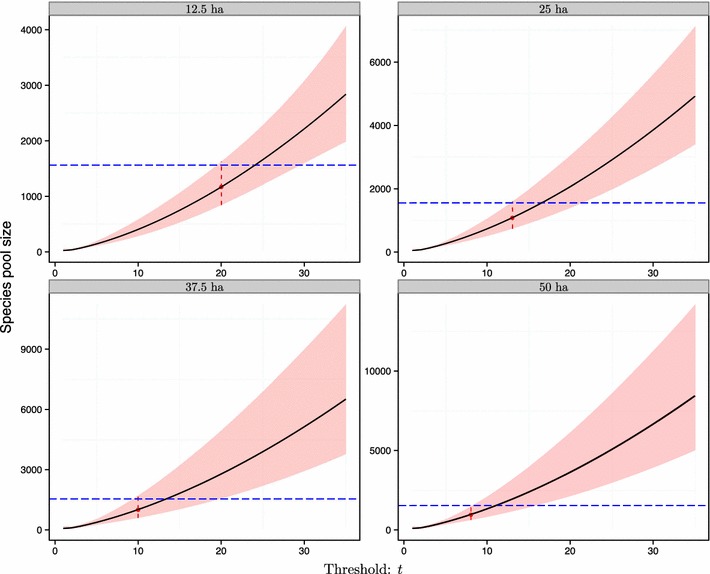
Relationships between area size of the associated species pool and population rarity threshold for the BCI local forest plot. The shadowed area is the 95% confidence band of *A* when the corresponding threshold *t* varies. Four sampling fractions of the entire BCI plot (0.25: 12.5, 0.5: 25, 0.75: 37.5 and 1.0: 50 ha) were analyzed and compared for their consistency in estimating the species pool. The vertical dashed line indicates the optimal threshold identified from Fig. 2 for each sampling scale. The horizontal dashed line indicates the area size of the entire BCI island

The demarcation of the species pool is strongly related to the threshold of population rarity (Fig. 3). When the rarity threshold is set larger, more rare species are taken into account, and the estimated area size of the regional species pool is accordingly larger, regardless of the sampling fraction used (Fig. 3).

Based on this optimal threshold, our empirical test showed that the local species pool for the 50-ha local BCI tree community was around 1000 ha with 95% confidence interval bounds at 636.19 and 1504.89 when the entire BCI plot was sampled and analyzed (Table 1; Fig. 3). This is close to the area size of the entire BCI island (~ 1560 ha). Thus, we conclude BCI island is sufficient to be a local, if not regional species pool for the 50 ha BCI stem-mapping plot. Accordingly, the estimated species number of the pool was 360 with the 95% confidence interval bounds at 339 and 392. We note that the calculated species pool would be smaller if the sampling fraction was smaller.

For the four different sampling fractions of the entire BCI forest plot, although the pool area size estimation would decrease slightly with increasing sampling fraction (Table 1), the estimated species richness of the pool was uncorrelated with sampling scale (Table 1). More importantly, the 95% confidence intervals of species richness and area size for different sample scales overlapped extensively.

Finally, in addition to the empirical test shown above, the numerical test further demonstrated the power and reliability of the proposed method in estimating species pools. The true values of area size (i.e., *A* = 1500) and species richness (i.e., *S*_*A*_ = 2000) for the hypothetical species pool have been covered very well by the 95% confidence intervals when the optimal thresholds were determined by the suggested procedure using Eq. (7) (Figs. S2 and S3, and Table S1 of Additional file 1) regardless of the sampling scales studied.

## Discussion

### Advantages of applying the truncated NTD model

The present study provides a simple probabilistic method for delineating the area size and estimating species richness of the species pool for a local ecological community in which limited species abundance information is available. One key novelty of our model is that it is unnecessary to know or estimate species richness (*S*_*A*_) of the pool when estimating the pool areal size (*A*). The truncated NTD model (Eq. 1) and its marginal distribution Eq. (2) contains the information for pool size *A,* which could be estimated using Eq. (6). Of course, the species number presented in the pool could be easily estimated after the area size for the pool has been estimated using Eq. (9).

Importantly in the present model, the size of the pool is presumably related to the distributional aggregation of species. If more species have aggregated distributions, it is likely that there are more rare species present in the pool but not observed in the local samples. In this case, the rarity threshold *t* should be set higher and as a result, the area size of the pool should be larger (Table 1). The employed model in Eq. (1) or (2) is an extension of the NBD model and can depict the possible spatial distributional patterns of species in the local community because the NBD is quite general. Therefore, the model can characterize diverse patterns of species spatial distribution, including aggregation, regularity and randomness [29, 30, 41, 42].

Another key point in our assumption is that the species pool represents a large ecological community containing all species that can colonize the local community or remaining part of the pool. The truncated NTD (Eq. 1) reflects this assumption, as it would return zero if a species is not present in either part of the pool (i.e., *a* or *h*). Consequently, such species would be excluded in the estimation of species pool size and richness; moreover, any species from the pool should be present in the local community with positive probabilistic values. The marginal probability of the truncated NTD can reflect this fact, as the probability of a species presence in the pool using Eq. (2) is never zero when *a* = *A*; that is, the absence probability using Eq. (2) in the pool *A* is zero. In contrast, the absence of a species could be possible in a local area *a* when *a* < *A*. Lastly, its marginal distribution allows the species pool area size *A* to enter Eq. (2) directly, which is required to be independently estimated when other parameters (*α* and *β*) have been estimated in advance.

### Information provided by unseen and rare species on local or regional species pools

Based on the original definition, a species pool should only contain those species that can colonize or recolonize the local site readily when environmental or habitat conditions have changed. In classical richness estimators, unseen species represent the species that have not been seen in the local site at the current time, but would become detectable if more extensive field surveys are conducted in the local site or the sampling area is expanded to include neighboring areas.

Nearly all richness estimators, such as Jackknife, Chao and others [26, 37, 38, 43, 44], have incorporated species with single or double individuals in the ecological community to estimate the lower bound of the number of unseen species in the community. However, these low-bound richness estimators rarely consider the information of other rare or even common species, and an exception case is the bootstrap estimator [45].

However, for estimating species pool area size in our model, in addition to singleton and doubleton species, we further considered other rare species (not as so rare as the singletons or doubletons, but rare enough, such as species with three, four or five individuals, etc., defined by a rarity threshold (Eq. 4). The key reason for inclusion of other less rare species from the local community is based on the fact that unseen species in sampled area *a* (Fig. 1) would be those species that occur in the unsampled region *h* with low abundances. As mentioned previously, these species in *h* with small abundances 1, 2, …, *t* would be very likely to be unseen in the sampled area *a*, contributing to the estimation of unseen species in the sampled area. As a result, we hypothesize that the threshold of rarity is dynamically related to the number of unseen species when the local area size *a* varies.

To define an optimal threshold value of rarity, our method considers that there is a tradeoff between the changing rates of *P*(*X*_*a*_ = 0) and *P*(*X*_*h*_ = 0), when the species pool size *A* increases (Eq. 7). This tradeoff is based on the premise that when the pool is sufficiently large, the numbers of unseen species in both sampled site *a* and unsampled habitat *h*, respectively would reach stable values that will not change or will change slowly, no matter how *A* is further expanded. Because we have two unknown variables *t* and *h*, we were able to solve them using both Eqs. (6) and (7). The other parameters, *α* and *β*, describing spatial distribution have been estimated independently of *t* and *h* using Eq. (3b).

Conclusively, our present method is not simply a classic richness estimator in comparison to previous studies [26, 38, 45]. Unlike previous richness estimators, our method incorporated the abundance information of rare species in the truncated NTD model. To this end, our method is more suitable for estimating the areal size or extrapolating species number of a species pool covering a vast area, even at a broad biogeographic scale (as demonstrated in the numerical test, the ratio between the area sizes for the pool and the target community is *A*/*a* = 25: Figs. S1–S3 and Table S1 of Additional file 1). This is accomplished using species abundance information from a local ecological community at a very small spatial scale. A recent review paper [46] also evaluated different methods, taking Hui’s Occupancy Rank Curve for instance [47], for conducting broad-scale richness extrapolation from local spatial scales. However, the exceptional advantage of our proposed method is that it can estimate optimal species richness and area size simultaneously, both of which are indispensible components for defining species pools.

### Robustness of our model with respect to sample size

**Fig. 4:**
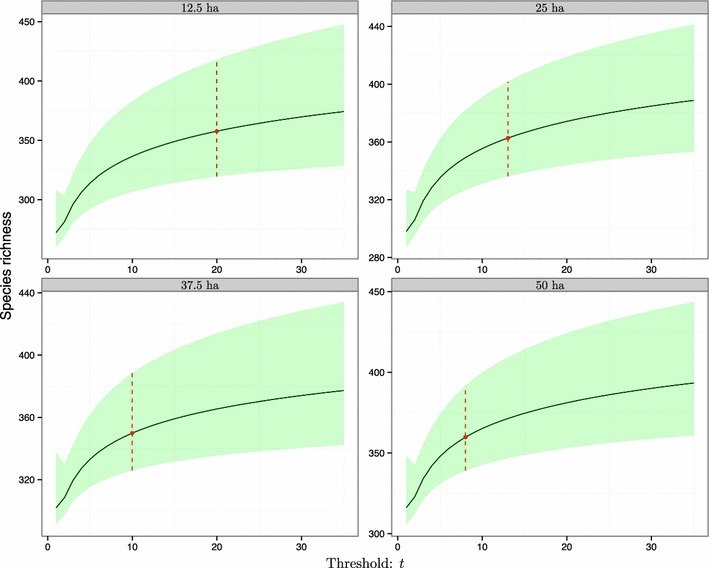
Relationships between estimated species richness ($S_{{\hat{A}}}$) for the associated species pool and population rarity threshold for the BCI local forest plot. The shadowed area is the 95% confidence band for *S*_*A*_ when the corresponding threshold *t* varies. Four sampling fractions of the entire BCI plot, as shown in Fig. 3, were analyzed and compared for their consistency in estimating regional species richness. The vertical dashed line indicates the optimal threshold identified from Fig. 2 for each sampling scale

If only a part of the entire BCI forest plot was sampled, the estimation of species diversity and area size for the species pool would not be altered. This is because our method is insensitive to changing sampling size (Table 1 and Figs. 3, 4). The 95% confidence intervals for species richness and area size, respectively, under different sampling fractions (or resultant local areas) would largely overlap from each other (Table 1). When the sampling fraction was 0.25, the estimation of the 95% confidence interval for the species richness for the pool was bounded by 319.0 and 417.7. The upper bound was close to the flora record of the number of tree species (including shrubs, around 450) on the island [48, 49]. The results for the numerical example further strongly proved that our method is insensitive to changing sampling size (Figs. S2, S3 and Table S1 of Additional file 1): no matter what the local sampling fraction is, the estimated area size and species number for the hypothetical species pool are always close to the true values (Table S1 of Additional file 1); and of course, the corresponding true values are well covered by the 95% confidence intervals of both research targets (Figs. S2, S3 and Table S1 of Additional file 1).

The key reason that the estimation using our method is consistent across different sampling scales is the use of the optimal threshold. When the sampling fraction of the entire forest plot was larger, the optimal rarity threshold *t* would decrease (Table 1). This is reasonable: as more areas of the entire species pool have been sampled (i.e., sampling fraction of the area *a* increases), fewer unseen species are expected in the remaining habitat *h* of the pool. Thus, only those rare species with extremely small population sizes hidden in *h* would be unseen when conducting species surveys in *a*. In such a case, the rarity threshold *t* is expected to be smaller.

### Comparison with other methods

As mentioned earlier, some previous studies [2, 18–20] also developed statistical methods to delineate the species pool. Most of these methods are probabilistic, similar to the method used in the present study. Moreover, akin to the rarity threshold used in our study, some methods [18, 20] utilized some kind of probability threshold to exclude or include species from the pool. However, other methods typically require abundance or incidence information (e.g., range maps) of species occurring in many local communities sampled from a broad spatial extent to compute community dissimilarity or measure range overlaps. Therefore, these methods are not applicable when only a single local community is sampled and studied.

Of course, it is necessary to mention that our method, the truncated NTD model used here, is parametric. The power of such parametric models concerning the estimation of species richness in the species pools depends on whether the local observed data satisfy the assumptions underlying the NTD or NBD (the marginal distribution of NTD) [50, 51]. However, as mentioned previously, because NBD or NTD models are very flexible on modeling species distribution ranging from random to highly aggregate patterns [51–53], it is of high likelihood that our model works very well in the estimation of species pool as to both area size and species richness.

## Conclusions

As a comparison and conclusion, our method for estimating local or regional species pool is quite simple and the only information needed is the species abundance information in the local sample. By incorporating the information from unseen and rare species, our method can provide diverse information, including an estimation of the species pool area size with statistical confidence intervals, evaluation of overall species distributional aggregation in the local community, estimation of species number in the pool, and number of unseen species that have been unobserved in the local community relative to its pool.

## Additional files

**Additional file 1.** Additional methods, figures and tables.

**Additional file 2.** R code for applying the proposed method to the estimation of species pools.
