# Supplementary material for: Estimating species pools for a single ecological assemblage
Source: BMC Ecol. 2017 Dec 22;17:45. doi: 10.1186/s12898-017-0155-7 (PMC5741966; doi:10.1186/s12898-017-0155-7)
Supplement: Supplementary file 1 — Additional file 1. Additional methods, figures and tables. [file 12898_2017_155_MOESM1_ESM.doc]

***Additional file 1***

**Estimating species pools for a single ecological assemblage**

Tsung-Jen Shen^1^, Youhua Chen^2,3*^, You-Fang Chen^4^

1, Institute of Statistics & Department of Applied Mathematics, National Chung Hsing University, 250 Kuo Kuang Road, Taichung, 40227 Taiwan

2, Chengdu Institute of Biology, Chinese Academy of Sciences, Chengdu, 610000, China

3, Department of Renewable Resources, University of Alberta, Edmonton, Alberta, T6G 2H1 Canada

4, School of Software, Harbin Normal University, Harbin, China

*Email for correspondence: [haydi@126.com](mailto:haydi@126.com)

**Additional methods**

*Detailed explanation of Eq. 1 in the main text*

Given $X_{A}=X_{a}+X_{h}=x+y>0$ (the total number of individuals of any species inhabiting the whole area *A*) and assume that the conditional distribution of ($X_{a}$,$X_{h}$) follows a binomial distribution with total $x+y$ and probability $\frac{a}{A}$, then we can express Eq. 1 of the main text by

$\begin{matrix} P(X_{a}=x,\text{ }X_{h}=y) \\ = P(X_{a}=x,\text{ }X_{h}=y|X_{a}+X_{h}=x+y)P(X_{a}+X_{h}=x+y) \\ = \left(\begin{matrix} x+y \\ x \end{matrix}\right)\left(\frac{a}{A}\right)^{x}\left(\frac{h}{A}\right)^{y}\times\frac{\Gamma(\alpha+x+y)}{\Gamma(x+y+1)\Gamma(\alpha)}\times\frac{\left(\frac{\beta}{\beta+A}\right)^{\alpha}\left(\frac{A}{\beta+A}\right)^{x+y}}{1-\left(1+A/\beta\right)^{-\alpha}} \\ = \frac{\Gamma(\alpha+x+y)}{\Gamma(x+1)\Gamma(y+1)\Gamma(\alpha)}\times\frac{\left(\frac{\beta}{\beta+A}\right)^{\alpha}\left(\left(\frac{a}{\beta+A}\right)^{x}\left(\frac{h}{\beta+A}\right)^{y}-I(x+y=0)\right)}{1-\left(1+A/\beta\right)^{-\alpha}} \end{matrix}$.

Note that the function of the conditional distribution $P(X_{a}=x,\text{ }X_{h}=y|X_{a}+X_{h}=x+y)$ is to distribute *x* individuals in the sampled area and the reminder *y* individuals in the unsampled area *h*. In a word, the numbers of individuals present in the sampled area *a* and in the unsampled area *h* are related to the relative area sizes $\frac{a}{A}$ and $\frac{h}{A}$, respectively.

In fact, from the definition of a binary variable $I(\cdot)$, we explicitly have $I(x+y=0)=1$ when $x=y=0$ and 0, otherwise (i.e., at least one of *x* and *y* is nonzero). Consequently, based on this clarification, Eq. 1 in the main text can be further explained by adding one more expression as

$\begin{matrix} P(X_{a}=x,\text{ }X_{h}=y) \\ = \frac{\Gamma(\alpha+x+y)}{\Gamma(x+1)\Gamma(y+1)\Gamma(\alpha)}\times\frac{\left(\frac{\beta}{\beta+A}\right)^{\alpha}\left(\left(\frac{a}{\beta+A}\right)^{x}\left(\frac{h}{\beta+A}\right)^{y}-I(x+y=0)\right)}{1-\left(1+A/\beta\right)^{-\alpha}} \\ = \left\{\begin{matrix} \frac{\Gamma(\alpha+x+y)}{\Gamma(x+1)\Gamma(y+1)\Gamma(\alpha)}\times\frac{\left(\frac{\beta}{\beta+A}\right)^{\alpha}\left(\left(\frac{a}{\beta+A}\right)^{0}\left(\frac{h}{\beta+A}\right)^{0}-1\right)}{1-\left(1+A/\beta\right)^{-\alpha}}=0,\text{ }x=y=0 \\ \frac{\Gamma(\alpha+x+y)}{\Gamma(x+1)\Gamma(y+1)\Gamma(\alpha)}\times\frac{\left(\frac{\beta}{\beta+A}\right)^{\alpha}\left(\frac{a}{\beta+A}\right)^{x}\left(\frac{h}{\beta+A}\right)^{y}}{1-\left(1+A/\beta\right)^{-\alpha}},\text{ otherwise}. \end{matrix}\right. \end{matrix}$.

Specifically, the joint probability distribution of $X_{a}$ and $X_{h}$ is truncated at $X_{a}+X_{h}=0$ or equivalently at $X_{a}=X_{h}=0$ since the top part of the second equality of the last equation is equal to zero as *x* = *y* =0 while the bottom part fulfills the theoretical property that its sum over all *x* and *y* equals one when being a probability distribution.

*Asymptotic variances for the estimated area size and species number of the species pool*

Since $A=a+h$ and *a* is fixed, the variance of the estimated area size *A*, found from Eq. (6) in the main text, is equal to the variance of the estimated *h* (i.e., $Var(\hat{A})=Var(\hat{h}$), which could be computed as: Define

$G(h,\hat{\alpha},\hat{\beta})=\left.\left\{\left(\left|P(X_{a}=0)-\sum_{i=1}^{t} P(X_{h}=i)\right|^{2}\right)^{\frac{1}{2}}+\left(\left|P(X_{h}=0)-\sum_{i=1}^{t} P(X_{a}=i)\right|^{2}\right)^{\frac{1}{2}}\right\}\right|_{(\alpha,\beta)=(\hat{\alpha},\hat{\beta})}$, (S1)

thus an estimate of *h*, $\hat{h}$, from Eq. (6) is equivalent to make . Using the Taylor expansion of at $\hat{h}=h$ gives

,

so we can approximate the variance of $\hat{h}$ by

, (S2)

which is Eq. 10 in the main text. Here, is estimated by

,  **(S3)**

where and $\Sigma$ is the observed information matrix with respect to the log likelihood function in Eq. (3b) and specifically expressed by

. **(S4)**

Regarding the variance of $S_{\hat{A}}$, we define which only involves , $\hat{\alpha}$, and $\hat{\beta}$ while it is unrelated to observed species richness $S_{a}$ in the sampled area. Using the variance decomposition formula gives

(S5)

which approximates

. (S6)

Conditional on $\hat{h}$, we apply the variance decomposition formula once again to as

(S7)

which approximates

, (S8)

where . Accordingly, the variance of $S_{\hat{A}}$ can be estimated by

(S9)

which is Eq. 11 in the main text.

*Theorem 1: Let the sampled data be ($Q_{1}$,$Q_{2}$,,$Q_{M}$) from the local area a, where $Q_{n}$ represents the number of species with n individuals in the data. When the observed number of species in the sampled area a is given, ($Q_{1}$,$Q_{2}$,,$Q_{M}$) follows a multinomial distribution.*

Proof: To show this, based on the sampled data in the area *a*, the likelihood function can be expressed as

$\begin{matrix} L(\alpha,\beta,S_{A}\left|Q_{1},...,Q_{M}\right.) =\frac{S_{A}!}{\prod_{k=0} Q_{k}!}\prod_{k=0} \left(P(X_{a}=k)\right)^{Q_{k}} \\ =\frac{S_{A}!}{Q_{0}!\left(S-Q_{0}\right)!}\left(P(X_{a}=0)\right)^{Q_{0}}\left(1-P(X_{a}=0)\right)^{S_{A}-Q_{0}}\times\\ \text{ }\frac{\left(\sum_{k=1}^{M} Q_{k}\right)!}{\prod_{k=1}^{M} Q_{k}!}\prod_{k=1}^{M} \left(\frac{P(X_{a}=k)}{1-P(X_{a}=0)}\right)^{Q_{k}} \end{matrix}$ (S10)

Note that the likelihood function in the second equality of Eq. S10 is composed of two parts. The first part is a binomial distribution with total $S_{A}$ and success probability $P(X_{a}=0)$ but involves the unknown parameter $S_{A}$ or is equivalently associated with the number of unseen species, $Q_{0}$, in the sampled area *a*. By contrast, the second part is only related to the parameters $\alpha$ and $\beta$ instead of being related to the parameter $S_{A}$. Since we only have observed data ($Q_{1}$,$Q_{2}$,,$Q_{M}$) in hand, our log likelihood function in Eq. 3b of the main text is based on the second part, which can be recognized as a standard multinomial distribution formula with total $\sum_{k=1}^{M} Q_{k}$ and cell probabilities , where . Using such a likelihood function to estimate parameters of interest is very common in literature (Mingoti & Meeden, 1992; Shen & He, 2008).

References

**Mingoti S, Meeden G**. **1992**. Estimating the total number of distinct species using presence and absence data. *Biometrics* **48**: 863–875.

**Shen T, He F**. **2008**. An incidence-based richness estimator for quadrats sampled without replacement. *Ecology* **87**: 2052–2060.

**Additional figures and tables**

Fig. S1. Square of the difference between the changing rates of unseen probabilities in the sampled area *a* and unsampled habitat *h*, for threshold *t* values for different sampling sizes in the hypothetical example. We choose a cutoff point here as $10^{-10}$ (shown as the black horizontal dashed line), after which the square of the difference would approach zero and thus decrease very slowly. Different curves represent different sampling fractions (0.25, 0.5, 0.75 and 1, or correspondingly, local area sizes 15, 30, 45 and 60) of the entire hypothetical local area *a* = 60 and used to infer the area size of the regional species pool (*A*=1500). The optimal threshold positions for different sampling sizes are highlighted with vertical arrows.


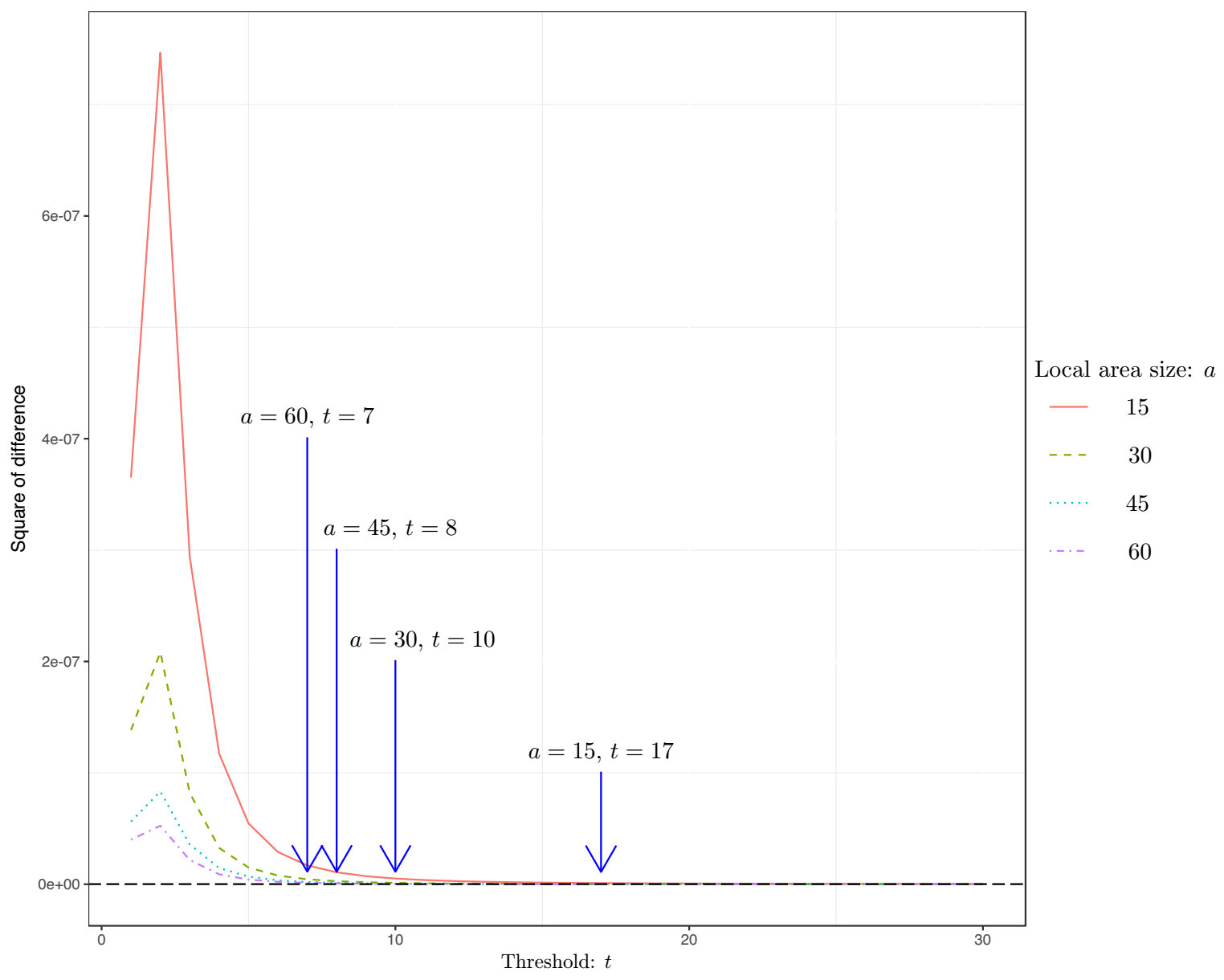


Fig. S2. Relationships between the estimated area size of the associated species pool and population rarity threshold for the hypothetical species pool example (true values: *A*=1500 and $S_{A}=2000$). The shadowed area is the 95% confidence band of *A* when the corresponding threshold *t* varies. Four sampling fractions (0.25, 0.5, 0.75 and 1) of the hypothetical local area *a* with size 60 were analyzed and compared for their consistency in estimating the species pool. The vertical dashed line indicates the optimal threshold identified from Fig. S1 for each sampling scale. The horizontal dashed line indicates the true area size *A* = 1500 of the hypothetical species pool.


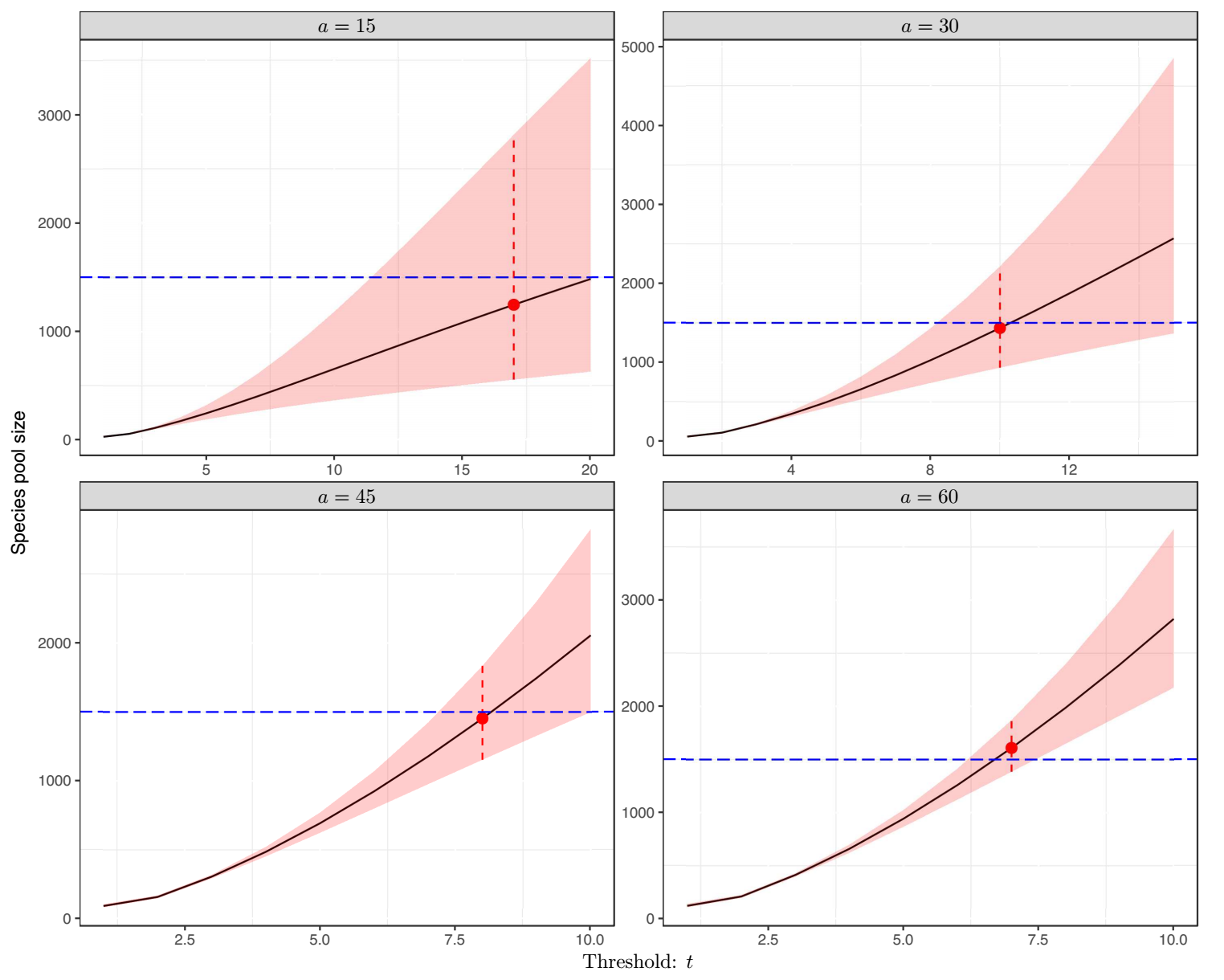


Fig. S3. Relationships between estimated species richness ($S_{\hat{A}}$) for the associated species pool and population rarity threshold for the hypothetical species pool. The shadowed area is the 95% confidence band for $S_{A}$ when the corresponding threshold *t* varies. Four sampling fractions (0.25, 0.5, 0.75 and 1) of the local area with size 60 were analyzed and compared for their consistency in estimating regional species richness. The vertical dashed line indicates the optimal threshold identified from Fig. S1 for each sampling scale. The horizontal dashed line indicates the true species richness $S_{A}=2000$ of the hypothetical species pool with size *A*=1500.


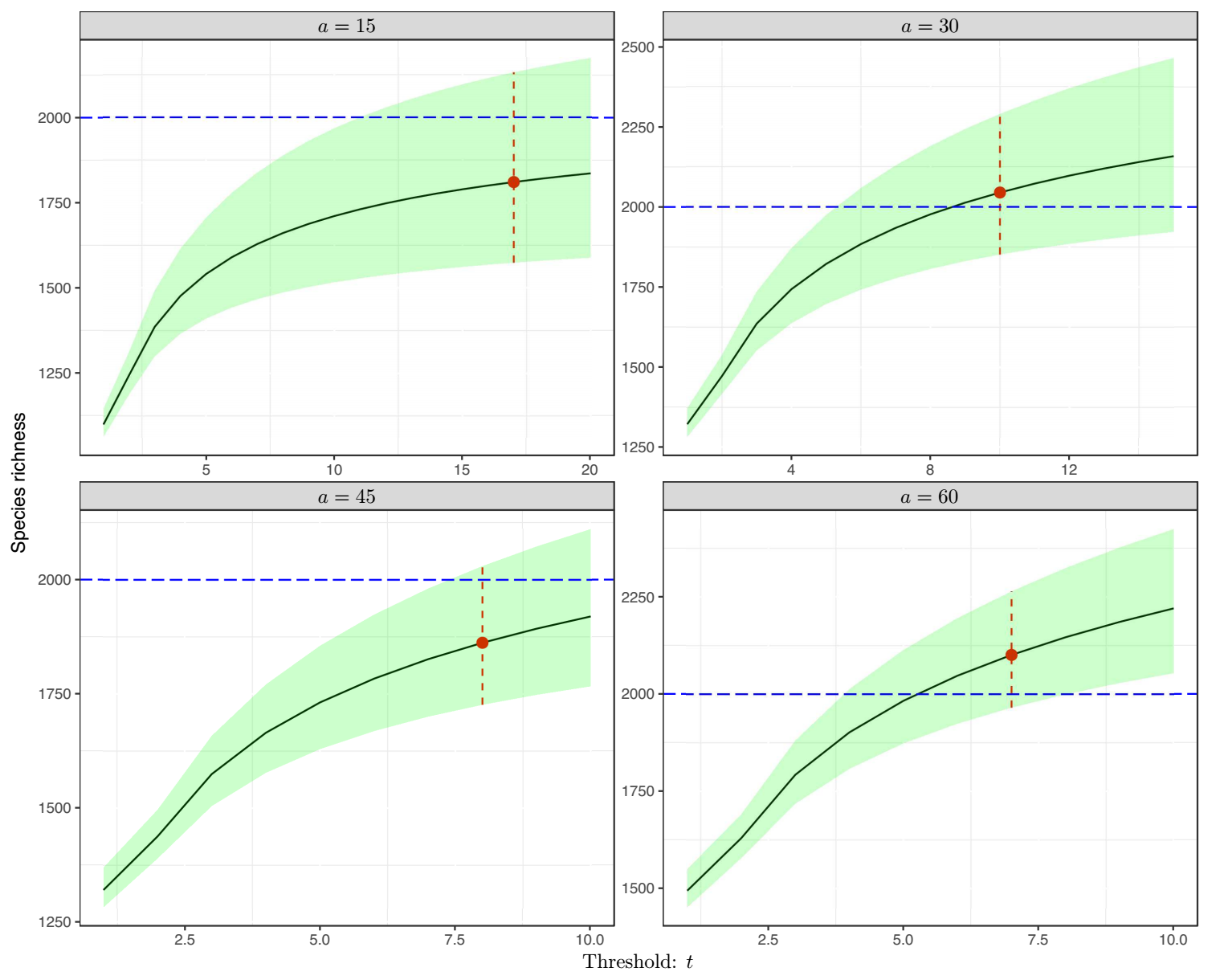


Table S1. Estimated area size, species richness, and 95% confidence intervals of the species pool for the hypothetical species pool with the true area size *A* = 1500 and the true species richness $S_{A}=2000$. Results from the four different sampling fractions are presented and compared to show the estimated robustness of our model with respect to varying sampling sizes. Optimal rarity threshold *t* for each sampling scale is also provided for reference.

| Sampling fraction | Optimal threshold *t* | Area size of pool *A* | |  | Species richness of pool $S_{A}$ | |
| --- | --- | --- | --- | --- | --- | --- |
|  |  | Estimate | 95% CI |  | Estimate | 95% CI |
| 0.25 | 17 | 1247.1 | (557.5, 2813.4) |  | 1810.2 | (1574.0, 2131.7) |
| 0.50 | 10 | 1435.1 | (933.3, 2215.6) |  | 2044.7 | (1851.4, 2289.7) |
| 0.75 | 8 | 1453.1 | (1153.0, 1834.5) |  | 1862.1 | (1726.3, 2030.1) |
| 1.0 | 7 | 1609.0 | (1384.6, 1871.3) |  | 2101.0 | (1965.3, 2264.6) |
