# Supplementary material for: Estimating species pools for a single ecological assemblage
Source: BMC Ecol. 2017 Dec 22;17:45. doi: 10.1186/s12898-017-0155-7 (PMC5741966; doi:10.1186/s12898-017-0155-7)
Supplement: Supplementary file 2 — Additional file 2. R code for applying the proposed method to the estimation of species pools. [file 12898_2017_155_MOESM2_ESM.doc]

***Additional file 2***

**Estimating species pools for a single ecological assemblage**

Tsung-Jen Shen^1^, Youhua Chen^2,3*^, You-Fang Chen^4^

1, Institute of Statistics & Department of Applied Mathematics, National Chung Hsing University, 250 Kuo Kuang Road, Taichung, 40227 Taiwan

2, Chengdu Institute of Biology, Chinese Academy of Sciences, Chengdu, 610000, China

3, Department of Renewable Resources, University of Alberta, Edmonton, Alberta, T6G 2H1 Canada

4, School of Software, Harbin Normal University, Harbin, China

*Email for correspondence: [haydi@126.com](mailto:haydi@126.com)

*R code for applying the proposed method to estimate species pools*

Our R code includes five steps described below that demonstrate how to use the proposed method to estimate the species pool regarding its area size along with species richness. To easy recognize our descriptions and R scripts, hereafter the R scripts along with detailed notations are in blue. As a suggestion, the R scripts of the four steps can be saved as five different files separately, and then sequentially being executed.

**Step 1: the following block of R scripts is executed to simulate data first.**

rm(list=ls())

library(nlme)

#### The pmf of the TNTD (Truncated Negative Trinomial Distribution); Eq. 2 of the main text

TNTD.fun = function(Xi,k,omega,a,A){

p=omega/(omega+a)

deno = 1-(omega/(A+omega))^k

if(Xi==0) {

pmf = ((omega/(a+omega))^k-(omega/(A+omega))^k)/deno

} else {

pmf = dnbinom(Xi,size=k,prob=p)/deno

} # else

pmf

}

### Species abundance data gererated by TNTD

RN.TNTD=function(n,a,A=1,shape,rate)

{

Y=rep(0, n)

for(i in 1:n)

{

u2=runif(1)

j=0; pr=TNTD.fun(0,k=shape,omega=rate,a,A); F=pr

if(u2<F){Y[i]=j}

else{

repeat{

pr=TNTD.fun(j+1,k=shape,omega=rate,a,A)

j=j+1; F=F+pr

if(u2<F) break

}

Y[i]=j

}

}

Y

}

## Find the MLEs of alpha and beta from the likelihood function,

## which is Eq. 3b of the main text

############### MLE #################

cond.logf <- function(x, f, m=1) {

a <- x[1]

b <- x[2]

pp = b/(b + m)

zz = which(f > 0)

rhoN = lgamma(zz + a) - lgamma(zz + 1) - lgamma(a) + zz *

log1p(-pp) + a * log1p(pp - 1) - log(1 - pp^a)

res <- -sum(f[zz] * rhoN)

res

}

############### MLE (end) #################

#(A,loacl.a)=(2000,100);(beta,k)=(1,0.08)

#(A,loacl.a)=(2000,100);(beta,k)=(0.8,0.08)

set.seed(2017)

## Setting the true species richness in the species pool

S = 2000

## Setting the true area size of the species pool

A = 1500

### Setting the true parameters in Eq. 2 of the main text

beta=1 ## for the parmeter beta

k= 0.05 ## for the parameter alpha

local.a =60

## When numerically solving the MLE of beta and alpha,

## we set a lower limit for both parameters

alpha.lower = 1e-10

### We set the local area size is 60

####### 100%

area = local.a

Xi = RN.TNTD(S,area,A,shape=k,rate=beta)

dimnames(Xi) = NULL

f = factor(Xi, levels = 0:max(Xi))

f = table(f, exclude = 0) ## frequency counts

dimnames(f) = NULL

cond.logfSol = nlminb(c(0.02, 0.003), cond.logf, f = f, m=area,

lower = c(alpha.lower, alpha.lower), upper = c(1000, 1000))

cond.ahat = cond.logfSol$par[1]

cond.bhat = cond.logfSol$par[2]

Fisher.Info = (fdHess(c(cond.ahat,

cond.bhat), f = f, m = area, fun = cond.logf)$Hessian)

k = cond.ahat

b = cond.bhat

save(area,f,k,b,Fisher.Info,

file=paste0("./SimulationStudy/SimData_100.rdata"))

### We set the local area size is 75% of 60

####### 75%

area = local.a*0.75

Xi = RN.TNTD(S,area,A,shape=k,rate=beta)

dimnames(Xi) = NULL

f = factor(Xi, levels = 0:max(Xi))

f = table(f, exclude = 0) ## frequency counts

dimnames(f) = NULL

cond.logfSol = nlminb(c(0.02, 0.003), cond.logf, f = f, m=area,

lower = c(alpha.lower, alpha.lower), upper = c(1000, 1000))

cond.ahat = cond.logfSol$par[1]

cond.bhat = cond.logfSol$par[2]

Fisher.Info = (fdHess(c(cond.ahat,

cond.bhat), f = f, m = area, fun = cond.logf)$Hessian)

k = cond.ahat

b = cond.bhat

save(area,f,k,b,Fisher.Info,

file=paste0("./SimulationStudy/SimData_75.rdata"))

### We set the local area size is 50% of 60

####### 50%

area = local.a*0.5

Xi = RN.TNTD(S,area,A,shape=k,rate=beta)

dimnames(Xi) = NULL

f = factor(Xi, levels = 0:max(Xi))

f = table(f, exclude = 0) ## frequency counts

dimnames(f) = NULL

cond.logfSol = nlminb(c(0.02, 0.003), cond.logf, f = f, m=area,

lower = c(alpha.lower, alpha.lower), upper = c(1000, 1000))

cond.ahat = cond.logfSol$par[1]

cond.bhat = cond.logfSol$par[2]

Fisher.Info = (fdHess(c(cond.ahat,

cond.bhat), f = f, m = area, fun = cond.logf)$Hessian)

k = cond.ahat

b = cond.bhat

save(area,f,k,b,Fisher.Info,

file=paste0("./SimulationStudy/SimData_50.rdata"))

### We set the local area size is 25% of 60

####### 25%

area = local.a*0.25

# A = 1000*area

Xi = RN.TNTD(S,area,A,shape=k,rate=beta)

dimnames(Xi) = NULL

f = factor(Xi, levels = 0:max(Xi))

f = table(f, exclude = 0) ## frequency counts

dimnames(f) = NULL

cond.logfSol = nlminb(c(0.02, 0.003), cond.logf, f = f, m=area,

lower = c(alpha.lower, alpha.lower), upper = c(1000, 1000))

cond.ahat = cond.logfSol$par[1]

cond.bhat = cond.logfSol$par[2]

Fisher.Info = (fdHess(c(cond.ahat,

cond.bhat), f = f, m = area, fun = cond.logf)$Hessian)

k = cond.ahat

b = cond.bhat

save(area,f,k,b,Fisher.Info,

file=paste0("./SimulationStudy/SimData_25.rdata"))

**Step 2: the following block of R scripts is executed to estimate the area size and species richness of the hypothetical species pool. In addition, information of the corresponding 95% confidence bands regarding *A* and $S_{A}$ is stored in four preset files based on four sampling scales.**

rm(list = ls()) # Careful! This clears all of R's memory!

library(nlme)

#### Eq. 2

pmf.TNTD = function(max.x, k, b, a, h) {

pi = rep(0, max.x)

for (i in 1:max.x) {

pi[i] = gamma(k + i)/gamma(i + 1)/gamma(k) * (b/(b +

a))^k * (a/(b + a))^i/(1 - (1 + (h + a)/b)^(-k))

}

p0 = (b/(b + a))^k * (1 - ((b + a)/(b + h + a))^k)/(1 - (1 + (h + a)/b)^(-k))

list(p0 = p0, pi = pi)

}

### Eq. 6

d <- function(max.x, k, b, a, h) {

pmf.a = pmf.TNTD(max.x, k, b, a, h)

pmf.h = pmf.TNTD(max.x, k, b, h, a)

obj = (pmf.a[[1]][1]-sum(pmf.h[[2]][1:max.x]))^2+(pmf.h[[1]][1]-sum(pmf.a[[2]][1:max.x]))^2

# obj = abs((pmf.a[[1]][1]-sum(pmf.h[[2]][1:max.x])))+abs((pmf.h[[1]][1]-sum(pmf.a[[2]][1:max.x])))

return(obj)

}

#### Find an optimal solution of h

Sp.pool = function(max.x, k, b, a) {

res = optimize(d, lower = (a * 1.00001-a), upper = 1e+10 * a,

tol = 1e-04, max.x = max.x, k = k, b = b, a = a)

res

}

### Eq. 7

## Here the two partial derivatives with respect to the two probabilities

## are numerically evaluated

optimal<-function(x,k,b,a)

{

diff = 10^(-5)

max.x=1

h=x[2]

pmf.a=pmf.TNTD(max.x,k,b,a,h)

pmf.h=pmf.TNTD(max.x,k,b,h,a)

#

AA = pmf.TNTD(max.x,k,b,a,h+diff)[[1]]

BB = pmf.TNTD(max.x,k,b,h+diff,a)[[1]]

obj=(((AA-pmf.a[[1]])+(BB-pmf.h[[1]]))/diff)^2

return(obj)

}#

## The function G in the main text is used to calculate

## the variance of estimated h

G.fun = function(max.x, k, b, a, h) {

pmf.a = pmf.TNTD(max.x, k, b, a, h)

pmf.h = pmf.TNTD(max.x, k, b, h, a)

sqrt((pmf.a[[1]]-sum(pmf.h[[2]][1:max.x]))^2)+sqrt((pmf.h[[1]]-sum(pmf.a[[2]][1:max.x]))^2)

}

p.h.G.fun = function(diff = 1e-05, max.x, k, b, a, h) {

(G.fun(max.x, k, b, a, h + diff) - G.fun(max.x, k, b, a,

h))/diff

}

p.k.G.fun = function(diff = 1e-05, max.x, k, b, a, h) {

(G.fun(max.x, k + diff, b, a, h) - G.fun(max.x, k, b, a,

h))/diff

}

p.b.G.fun = function(diff = 1e-05, max.x, k, b, a, h) {

(G.fun(max.x, k, b + diff, a, h) - G.fun(max.x, k, b, a,

h))/diff

}

Var.h = function(diff = 1e-05, max.x, h, k, b, a, sigma) {

u = matrix(c(p.k.G.fun(diff, max.x, k, b, a, h), p.b.G.fun(diff,

max.x, k, b, a, h)), nrow = 1)

var.G = as.numeric(u %*% solve(sigma) %*% t(u))

AA = p.h.G.fun(diff, max.x, k, b, a, h)

var.G/AA^2

}

##### the following functions are used for

## the variance estimation of S_A

H.fun = function(k, b, a, h) {

pmf.a = pmf.TNTD(1, k, b, a, h)

1/(1-pmf.a[[1]])

}

p.h.H.fun = function(diff = 1e-05, k, b, a, h) {

(H.fun(k, b, a, h + diff) - H.fun(k, b, a,

h))/diff

}

p.k.H.fun = function(diff = 1e-05, k, b, a, h) {

(H.fun(k + diff, b, a, h) - H.fun(k, b, a,

h))/diff

}

p.b.H.fun = function(diff = 1e-05, k, b, a, h) {

(H.fun(k, b + diff, a, h) - H.fun(k, b, a,

h))/diff

}

#### Eq. 11

var.SA = function(diff = 1e-05, Sa, h, k, b, a, sigma, var.h, SA) {

u = matrix(c(p.k.H.fun(diff, k, b, a, h),

p.b.H.fun(diff, k, b, a, h)), nrow = 1)

var.H = as.numeric(u %*% solve(sigma) %*% t(u))

AA = var.h*(p.h.H.fun(diff, k, b, a, h))^2

VAR.H = var.H + AA

Var = Sa^2*VAR.H + H.fun(k, b, a, h)^2*Sa*(1-Sa/SA)

Var

}

########################################

########################################

# 100 percent

########################################

########################################

load("./SimulationStudy/SimData_100.rdata")

ObsS = sum(f)

Sa = ObsS

a = area

diff.val = 1e-5

Cut = 1:50

val.A = vector()

se.A=rep(0,length(Cut))

val2 = c()

est.SA=c()

se.S=c()

for (i in Cut) {

val.A[i] = as.numeric(Sp.pool(i, k, b, a)[1])+a

se.A[i] = sqrt(Var.h(diff.val, i, h=val.A[i]-a, k, b, a, Fisher.Info))

est.SA[i] = Sa*H.fun(k, b, a, h=val.A[i]-a)

se.S[i]=sqrt(var.SA(diff.val, Sa, val.A[i]-a, k, b, a, Fisher.Info, var.h=se.A[i]^2, est.SA[i]))

} #i

### the 95% CI of species pool size by Chao's logtransformation

S = val.A

D = a

f0 = S - D

var.S = se.A^2

R = exp(1.96 * (log(1 + (var.S)/(f0^2)))^0.5)

### the 95% CI of species richness of the pool by Chao's logtransformation

S.richness = est.SA

D.richness = Sa

f0.richness = S.richness - D.richness

var.S.richness = se.S^2

R.richness = exp(1.96 * (log(1 + (var.S.richness)/(f0.richness^2)))^0.5)

for(i in Cut) {

val2[i]=optimal(c(i,val.A[[i]][1]),k,b,a)

}#i

output100 = data.frame(diff=val2,t=Cut,se.A=se.A,

val.A=val.A,

log.lower = D + f0/R, log.upper = D + f0 * R,

log.lower.sp = Sa + f0.richness/R.richness,

log.upper.sp = Sa + f0.richness * R.richness,

se.S=se.S,est.SA=est.SA)

save(output100, file = "./SimulationStudy3/output_sim_100.Rdata")

########################################

########################################

# 75 percent

########################################

########################################

# load("./SubArea/bci_75_area.rdata")

load("./SimulationStudy/SimData_75.rdata")

ObsS = sum(f)

Sa = ObsS

a = area

diff.val = 1e-5

Cut = 1:50

val.A = vector()

se.A=rep(0,length(Cut))

val2 = c()

est.SA=c()

se.S=c()

for (i in Cut) {

val.A[i] = as.numeric(Sp.pool(i, k, b, a)[1])+a

se.A[i] = sqrt(Var.h(diff.val, i, h=val.A[i]-a, k, b, a, Fisher.Info))

est.SA[i] = Sa*H.fun(k, b, a, h=val.A[i]-a)

se.S[i]=sqrt(var.SA(diff.val, Sa, val.A[i]-a, k, b, a, Fisher.Info, var.h=se.A[i]^2, est.SA[i]))

} #i

### the 95% CI of species pool by Chao's logtransformation

S = val.A

D = a

f0 = S - D

var.S = se.A^2

R = exp(1.96 * (log(1 + (var.S)/(f0^2)))^0.5)

### the 95% CI of species pool by Chao's logtransformation

S.richness = est.SA

D.richness = Sa

f0.richness = S.richness - D.richness

var.S.richness = se.S^2

R.richness = exp(1.96 * (log(1 + (var.S.richness)/(f0.richness^2)))^0.5)

# plot(Cut, val.A, type = "l", ylab = "h (in ha)", xlab = "sum of rare species")

# abline(h=15.6/0.5*50-50,col="red")

for(i in Cut) {

val2[i]=optimal(c(i,val.A[[i]][1]),k,b,a)

}#i

output75 = data.frame(diff=val2,t=Cut,se.A=se.A,

val.A=val.A,

log.lower = D + f0/R, log.upper = D + f0 * R,

log.lower.sp = Sa + f0.richness/R.richness,

log.upper.sp = Sa + f0.richness * R.richness,

se.S=se.S,est.SA=est.SA)

save(output75, file = "./SimulationStudy3/output_sim_75.Rdata")

########################################

########################################

# 50 percent

########################################

########################################

load("./SimulationStudy/SimData_50.rdata")

ObsS = sum(f)

Sa = ObsS

a = area

diff.val = 1e-5

Cut = 1:50

val.A = vector()

se.A=rep(0,length(Cut))

val2 = c()

est.SA=c()

se.S=c()

for (i in Cut) {

val.A[i] = as.numeric(Sp.pool(i, k, b, a)[1])+a

se.A[i] = sqrt(Var.h(diff.val, i, h=val.A[i]-a, k, b, a, Fisher.Info))

est.SA[i] = Sa*H.fun(k, b, a, h=val.A[i]-a)

se.S[i]=sqrt(var.SA(diff.val, Sa, val.A[i]-a, k, b, a, Fisher.Info, var.h=se.A[i]^2, est.SA[i]))

} #i

### the 95% CI of species pool by Chao's logtransformation

S = val.A

D = a

f0 = S - D

var.S = se.A^2

R = exp(1.96 * (log(1 + (var.S)/(f0^2)))^0.5)

### the 95% CI of species pool by Chao's logtransformation

S.richness = est.SA

D.richness = Sa

f0.richness = S.richness - D.richness

var.S.richness = se.S^2

R.richness = exp(1.96 * (log(1 + (var.S.richness)/(f0.richness^2)))^0.5)

for(i in Cut) {

val2[i]=optimal(c(i,val.A[[i]][1]),k,b,a)

}#i

output50 = data.frame(diff=val2,t=Cut,se.A=se.A,

val.A=val.A,

log.lower = D + f0/R, log.upper = D + f0 * R,

log.lower.sp = Sa + f0.richness/R.richness,

log.upper.sp = Sa + f0.richness * R.richness,

se.S=se.S,est.SA=est.SA)

save(output50, file = "./SimulationStudy3/output_sim_50.Rdata")

########################################

########################################

# 25 percent

########################################

########################################

load("./SimulationStudy/SimData_25.rdata")

ObsS = sum(f)

Sa = ObsS

a = area

diff.val = 1e-5

Cut = 1:50

val.A = vector()

se.A=rep(0,length(Cut))

val2 = c()

est.SA=c()

se.S=c()

for (i in Cut) {

val.A[i] = as.numeric(Sp.pool(i, k, b, a)[1])+a

se.A[i] = sqrt(Var.h(diff.val, i, h=val.A[i]-a, k, b, a, Fisher.Info))

est.SA[i] = Sa*H.fun(k, b, a, h=val.A[i]-a)

se.S[i]=sqrt(var.SA(diff.val, Sa, val.A[i]-a, k, b, a, Fisher.Info, var.h=se.A[i]^2, est.SA[i]))

} #i

### the 95% CI of species pool by Chao's logtransformation

S = val.A

D = a

f0 = S - D

var.S = se.A^2

R = exp(1.96 * (log(1 + (var.S)/(f0^2)))^0.5)

### the 95% CI of species pool by Chao's logtransformation

S.richness = est.SA

D.richness = Sa

f0.richness = S.richness - D.richness

var.S.richness = se.S^2

R.richness = exp(1.96 * (log(1 + (var.S.richness)/(f0.richness^2)))^0.5)

for(i in Cut) {

val2[i]=optimal(c(i,val.A[[i]][1]),k,b,a)

}#i

output25 = data.frame(diff=val2,t=Cut,se.A=se.A,

val.A=val.A,

log.lower = D + f0/R, log.upper = D + f0 * R,

log.lower.sp = Sa + f0.richness/R.richness,

log.upper.sp = Sa + f0.richness * R.richness,

se.S=se.S,est.SA=est.SA)

save(output25, file = "./SimulationStudy3/output_sim_25.Rdata")

**Step 3: the following block of R scripts is executed to create a figure like Fig. 2 (the determination of the optimal rarity threshold *t* using Eq. 7 of the main text) in the main text but with the simulated data (therefore for Fig. S1 in Additional File 1).**

rm(list = ls())

library(nlme)

library("ggplot2")

library(reshape)

require(grid)

load("./SimulationStudy/output_sim_25.Rdata")

load("./SimulationStudy/output_sim_50.Rdata")

load("./SimulationStudy/output_sim_75.Rdata")

load("./SimulationStudy/output_sim_100.Rdata")

plot.data=data.frame(fraction=0.25,output25)

plot.data = rbind(plot.data,cbind(fraction=0.5,output50))

plot.data = rbind(plot.data,cbind(fraction=0.75,output75))

plot.data = rbind(plot.data,cbind(fraction=1,output100))

plot.data = subset(plot.data,t<=30)

plot.data$fraction = factor(plot.data$fraction)

pp = ggplot(plot.data, aes(x = t, y = diff, color=fraction))

pp = pp + geom_line(aes(lty=fraction))+

scale_linetype_manual(values=c(1:4))

data.cut = data.frame(c(fraction=0.25,output25[17,]))

data.cut = rbind(data.cut,c(fraction=0.5,output50[10,]))

data.cut = rbind(data.cut,c(fraction=0.75,output75[8,]))

data.cut = rbind(data.cut,c(fraction=1,output100[7,]))

pp = pp+geom_hline(yintercept=1e-10,colour="black",lty=5)

pp = pp+geom_segment(data=data.cut, mapping=aes(x=t, y=diff+1e-7*(1:4), xend=t, yend=diff+1e-8),

arrow=arrow(), size=.5, color="blue")

pp = pp+geom_text(data=data.cut, mapping=aes(x=t, y=diff+1e-7*((1:4)+0.17), label=LETTERS[1:4]),

size=5, color="blue")

pp = pp+ theme_bw()+xlab("Threshold")+ylab("Square of difference")+

theme(legend.position="right",

legend.text=element_text(color="black"),

legend.key.size = unit(1, "cm"),

legend.key= element_rect(linetype=0, color="white"),# lines around symbols

legend.key.width = unit(1., "cm"),

legend.margin = unit(.1, "cm"),

legend.direction="vertical",

plot.margin=unit(c(0.2,2,0.2,.2),"cm"),

panel.grid.major = element_line(colour = "white"),

panel.margin.x = unit(0.3, "lines"),

strip.text.x = element_text(colour = "black", angle = 0, size = 8,

hjust = 0.5, vjust = 0.5),

legend.title = element_text(color="black"))

print(pp)

ggsave("./SimulationStudy/Figure/Estimated_rate.eps",width = 10, height = 8, device=cairo_ps)

**Step 4: the following block of R scripts is to create a figure like Fig. 3 (estimation of species pool size) in the main text but with the simulated data (therefore for Fig. S2 in Additional File 1)**

rm(list = ls())

library(nlme)

library("ggplot2")

library(reshape)

require(grid)

load("./SimulationStudy/output_sim_25.Rdata")

load("./SimulationStudy/output_sim_50.Rdata")

load("./SimulationStudy/output_sim_75.Rdata")

load("./SimulationStudy/output_sim_100.Rdata")

plot.data=data.frame(fraction=0.25,output25)

plot.data = rbind(plot.data,cbind(fraction=0.5,output50))

plot.data = rbind(plot.data,cbind(fraction=0.75,output75))

plot.data = rbind(plot.data,cbind(fraction=1,output100))

plot.data1 = subset(plot.data,fraction==0.25 & t<=20)

plot.data1 = rbind(plot.data1, subset(plot.data,fraction==0.5 & t<=15))

plot.data1 = rbind(plot.data1, subset(plot.data,fraction==0.75 & t<=10))

plot.data1 = rbind(plot.data1, subset(plot.data,fraction==1 & t<=10))

plot.data=plot.data1

pp = ggplot(plot.data, aes(x = t, y = val.A))

pp = pp + geom_line()+

scale_linetype_manual(values=c(2))

pp= pp+geom_ribbon(data=plot.data,

aes(ymin=log.lower, ymax=log.upper, fill = "interval") ,alpha=0.2)+

scale_fill_manual("",values="red")

data.cut = data.frame(c(fraction=0.25,output25[17,]))

data.cut = rbind(data.cut,c(fraction=0.5,output50[10,]))

data.cut = rbind(data.cut,c(fraction=0.75,output75[8,]))

data.cut = rbind(data.cut,c(fraction=1,output100[7,]))

pp=pp+geom_pointrange(data=data.cut,size = 0.5,

aes(x = t, ymin = log.lower, ymax =log.upper, y = val.A),col="red",lty=2)

pp = pp+geom_hline(yintercept=1500,colour="blue",lty=5)

pp = pp+facet_wrap(~fraction, ncol=2,nrow=2,scales = "free")

pp = pp+ theme_bw()+xlab("threshold")+ylab("Species pool size")+

theme(legend.position="none",

legend.text=element_text(color="black"),

legend.key.size = unit(1, "cm"),

legend.key= element_rect(linetype=0, color="white"),# lines around symbols

legend.key.width = unit(1., "cm"),

legend.margin = unit(.1, "cm"),

legend.direction="vertical",

plot.margin=unit(c(0.2,.5,0.2,0.2),"cm"),

panel.grid.major = element_line(colour = "white"),

panel.margin.x = unit(0.3, "lines"),

strip.text.x = element_text(colour = "black", angle = 0, size = 8,

hjust = 0.5, vjust = 0.5),

legend.title = element_text(color="black"))

print(pp)

ggsave("./SimulationStudy/Figure/EstimatedArea.eps",width = 10, height = 8, device=cairo_ps)

**Step 5: the following block of R scripts is to create a figure like Fig. 4 (estimation of species richness in the species pool) in the main text but with the simulated data (therefore for Fig. S3 in Additional File 1)**

rm(list = ls())

library("ggplot2")

library(reshape)

require(grid)

load("./SimulationStudy/output_sim_25.Rdata")

load("./SimulationStudy/output_sim_50.Rdata")

load("./SimulationStudy/output_sim_75.Rdata")

load("./SimulationStudy/output_sim_100.Rdata")

plot.data=data.frame(fraction=0.25,output25)

plot.data = rbind(plot.data,cbind(fraction=0.5,output50))

plot.data = rbind(plot.data,cbind(fraction=0.75,output75))

plot.data = rbind(plot.data,cbind(fraction=1,output100))

plot.data1 = subset(plot.data,fraction==0.25 & t<=20)

plot.data1 = rbind(plot.data1, subset(plot.data,fraction==0.5 & t<=15))

plot.data1 = rbind(plot.data1, subset(plot.data,fraction==0.75 & t<=10))

plot.data1 = rbind(plot.data1, subset(plot.data,fraction==1 & t<=10))

plot.data=plot.data1

pp = ggplot(plot.data, aes(x = t, y = est.SA))

pp = pp + geom_line()+

scale_linetype_manual(values=c(2))

data.cut = data.frame(c(fraction=0.25,output25[17,]))

data.cut = rbind(data.cut,c(fraction=0.5,output50[10,]))

data.cut = rbind(data.cut,c(fraction=0.75,output75[8,]))

data.cut = rbind(data.cut,c(fraction=1,output100[7,]))

pp=pp+geom_pointrange(data=data.cut,size = 0.5,

aes(x = t, ymin = log.lower.sp, ymax =log.upper.sp, y = est.SA),col="red",lty=2)

pp= pp+geom_ribbon(data=plot.data,

aes(ymin=log.lower.sp, ymax=log.upper.sp, fill = "interval") ,alpha=0.2)+

scale_fill_manual("",values="green")

pp = pp+geom_hline(yintercept=2000,colour="blue",lty=5)

pp = pp+facet_wrap(~fraction, ncol=2,nrow=2,scales = "free")

pp = pp+ theme_bw()+xlab("threshold")+ylab("Species richness")+

theme(legend.position="none",

legend.text=element_text(color="black"),

legend.key.size = unit(1, "cm"),

legend.key= element_rect(linetype=0, color="white"),# lines around symbols

legend.key.width = unit(1., "cm"),

legend.margin = unit(.1, "cm"),

legend.direction="vertical",

plot.margin=unit(c(0.2,.5,0.2,0.2),"cm"),

panel.grid.major = element_line(colour = "white"),

panel.margin.x = unit(0.3, "lines"),

strip.text.x = element_text(colour = "black", angle = 0, size = 8,

hjust = 0.5, vjust = 0.5),

legend.title = element_text(color="black"))

print(pp)

ggsave("./SimulationStudy/Figure/Estimated_SpeciesRichness.eps",width = 10, height = 8, device=cairo_ps)
